# Supplementary material for: Novel dichloromethane-fermenting bacteria in the Peptococcaceae family
Source: ISME J. 2021 Jan 15;15(6):1709–21. doi: 10.1038/s41396-020-00881-y (PMC8163858; doi:10.1038/s41396-020-00881-y)
Supplement: Supplementary file 1 — Supplementary Information [file 41396_2020_881_MOESM1_ESM.pdf]

Supplementary Information for

Novel dichloromethane-fermenting bacteria in the *Peptococcaceae* family

Sophie I Holland, Haluk Ertan, Michael J Manefield, Matthew Lee

Supplementary Discussion

Necromass fermentation by cohabiting bacteria in culture DFE

Some of the most abundant cohabiting phylotypes in culture DFE – *Desulfovibrio*, *Bacteroidetes* (containing the taxa *Lentimicrobiaceae* and *Petrimonas*), *Spirochaetes/Treponematales*, *Synergistetes* – have previously been associated with hydrocarbon and organohalide-degrading mixed cultures (62–66), although their abundance was not linked to degradation of the primary substrate (with the exception of some *Desulfovibrio* species). Rather, some reports have suggested that these phylotypes persist via necromass recycling (16,65–67).

Of the cohabiting phylotypes in culture DFE, many have been shown to ferment compounds that would facilitate necromass recycling. *Synergistaceae* are well-known amino acid fermenters (68,69) and can be saccharolytic (70). It was expected they would be enriched with casamino acids, however they appear to have been outcompeted by *Veillonellaceae*. *Spirochaetes* are commonly detected in anoxic environments contaminated with hydrocarbons and organohalides (71,72) and a recent report demonstrated their role as necromass recyclers in such ecosystems (66). It was therefore expected that the *Synergistaceae* in culture DFE would be enriched with casamino acids, and the *Spirochaetes* with glucose, however they appear to have been outcompeted by the faster-growing *Veillonellaceae* and *Petrimonas* phylotypes, respectively (Fig. S4).

Members of the *Veillonellaceae* are typically associated with animal and/or human hosts and have been reported to ferment carbohydrates and metabolise organic acids and amino acids (73). *Petrimonas* and *Lentimicrobiaceae* are both members of the phylum *Bacteroidetes* with isolates shown to ferment a range of carbohydrates (74–76).

The sulphate-respiring genus *Desulfovibrio* has a broad substrate range and has been associated with contaminated environments both as a primary degrader (63,77) and a synergistic cohabitant consuming fermentation products of other organisms (18,65,72,78). A *Desulfovibrio* species is also present in an anaerobic DCM-dechlorinating culture dominated by *Dehalobacterium*, where it likely consumes formate produced from DCM metabolism and may also be capable of reducing minor amounts of sulphate present in the yeast extract added to the medium (36). Given that culture DFE is unable to consume exogenous formate and that the *Desulfovibrio* phylotype in culture DFE was enriched to apparent purity on peptone (Fig. S4C), it may persist here via a proteolytic metabolism.

Metabolism of choline by DCMF

When used as a substrate for growth, choline is typically metabolised into trimethylamine in anoxic subsurface environments (49,50,79), via a choline-trimethylamine lyase (CutC) (80). The inability of culture DFE to utilise exogenously provided trimethylamine and absence of this compound in choline-amended cultures suggested that a different metabolic pathway was being utilised here. Furthermore, the sole CutC homolog (Ga0180325_112585) in the DCMF genome contains only three of the six conserved residues predicted to be necessary for catalytic activity in other bacteria (80), is ~50 codons shorter than characterised CutC proteins, and not located within a bacterial microcompartment gene cluster.

Direct demethylation of choline to dimethylethanolamine has also been reported, although thus far only in methanogenic Archaea from the genus *Methanococcoides* (81). The enzyme catalysing this reaction is unknown but could reasonably be assumed to be a methyltransferase within the MttB superfamily, which DMCF encodes in abundance. Further stepwise demethylation of dimethylethanolamine would yield ethanolamine, which can be transformed to acetaldehyde and ammonium within a bacterial microcompartment, also encoded in the DCMF genome. However, as all nitrogen in the provided choline was recovered within MMA (127% ± 19% N recovery), this pathway seems less likely than transformation of choline to glycine betaine via betaine aldehyde in DCMF, as described in the main text.

Demethylation of glycine betaine

Glycine betaine (whether derived from choline or provided exogenously) is then likely demethylated, as has previously been reported in *Eubacterium limosum* (82), *Acetobacterium* spp. (83–85), and *Sporomusa* spp. (46). Demethylation is likely catalysed by a glycine betaine:corrinoid methyltransferase, encoded by a non-pyrrolysine member of the MttB superfamily (86). The glycine betaine:corrinoid methyltransferase (MT_1, MtgB) transfers a methyl group to a cognate corrinoid protein, and a methyl-tetrahydrofolate methyltransferase (MtgA, MT_2) then transfers the methyl group from the corrinoid protein to an accepting compound, tetrahydrofolate (86). In *S. ovata* strain An4, the same two *mtgB* genes were suggested to carry out demethylation of both glycine betaine and dimethylglycine, forming sarcosine (methylglycine) (47), whilst in *Acetobacterium woodii*, the protein appears to be specific to glycine betaine only, as there was no subsequent demethylation from dimethylglycine to sarcosine (87). MtgB homologs in the DCMF genome were previously described in a genome-based metabolic model, suggesting the organism may be capable of growth with glycine betaine and dimethylglycine (24), and an amended list of candidate proteins is included in Table S5, based

on homology to known glycine betaine methyltransferases. Whilst a dimethylglycine methyltransferase has not yet been conclusively described in the literature, the alternative dimethylglycine dehydrogenase enzyme could not be identified in the DCMF genome, lending support to the suggestion by Visser *et al* (47) of catalysis by a methyltransferase.

Reductive cleavage of sarcosine

The genetic potential for reductive cleavage of glycine betaine and sarcosine was also reported in this metabolic model (24). Presuming demethylation of glycine betaine to sarcosine, DFE cultures amended with sarcosine + H_2 were set up to help verify this metabolite as a pathway intermediate of choline and glycine betaine catabolism, as it could not be observed at any stage of growth. The production of methylamine, acetate, and DCMF cells was consistent the proposed pathway. The apparent ability of DCMF to utilise H_2 as an electron donor for reductive cleavage of sarcosine was at odds with its inability to grow with the classic acetogenic substrates H_2 + CO_2. The genome does encode a putative membrane-bound NiFe hydrogen uptake hydrogenase (HyaABCD, Ga0180325_111497-9, Ga0180325_111503) which may be utilised to provide reducing equivalents for the sarcosine reductase.

Given the presence of putative glycine betaine reductases in the genome (Ga0180325_115251 and Ga0180325_115252s54) (24), DFE cultures were then amended with glycine betaine and H_2 to test whether reductive cleavage of glycine betaine was also possible. However, trimethylamine was not produced, despite H_2 depletion (Fig. 2D). It is not yet clear which organisms in culture DFE were utilising the hydrogen, as controls amended only with hydrogen (i.e. glycine betaine-free or sarcosine-free) demonstrated no growth or acetogenesis. Concurrently, there was slightly higher acetate production observed in the glycine betaine + H_2 cultures (15 ± 0.6 mM, Fig. 2D), compared to the hydrogen-free glycine betaine cultures (11 ± 0.4 mM; Fig. 2C). It may be possible that CO_2 reduction by DCMF is enabled in the presence of glycine betaine and/or sarcosine, once other metabolic components (i.e. the WLP) are in use.

Theoretical energy balance for quaternary amine metabolism

Product formation and DCMF cell yields from the growth experiments with choline and glycine betaine were drawn together with the genomic information to generate a theoretical energy balance for consumption of choline and glycine betaine. The oxidation of two methyl groups from glycine betaine would yield 12 electrons (Eq. 6), of which two can be directed to reductive cleavage of sarcosine to yield one acetate and methylamine (Eq. 7). Given that sarcosine was not observed at any stage of growth, it is presumably rapidly cleaved. As eight electrons are required for acetate synthesis from bicarbonate (Eq. 8), the remaining 10 electrons equate to 1.25 acetate

equivalents via bicarbonate reduction (Eq. 9), totalling 2.25 mol acetate equivalents and 1 mol methylamine per mole glycine betaine (Eq. 10). This approximately accords with the observed acetate (2.3 ± 0.1 mM per mole glycine betaine) and methylamine (0.9 ± 0.1 mM per mole glycine betaine) concentrations in glycine betaine-amended cultures.

The methylamine yield in choline-amended cultures (1.3 ± 0.2 mM per mole choline utilised), was also close to the theoretical yield based on the above equations. The metabolism of choline into glycine betaine liberates four electrons (Eq. 11), which equate to an additional 0.625 mol acetate for each mol of choline metabolised to glycine betaine (Eq. 12). Combining the choline to glycine betaine, and glycine betaine to acetate and methylamine equations results in a theoretical yield of 2.75 mol acetate equivalents per mole choline (Eq. 13), which is within one standard deviation of the observed 3.1 ± 0.4 mM acetate per mole choline utilised.

$$(CH_3)_3N^+CH_2COO^- + 6\ H_2O \rightarrow (CH_3)NH_2^+CH_2COO^- + 2\ HCO_3^- + 14\ H^+ + 12\ e^- \qquad \text{Eq. 6}$$

$$(CH_3)NH_2^+CH_2COO^- + 2\ H^+ + 2\ e^- \rightarrow (CH_3)NH_3^+ + CH_3COO^- \qquad \text{Eq. 7}$$

$$2\ HCO_3^- + 9\ H^+ + 8\ e^- \rightarrow CH_3COO^- + 4\ H_2O \qquad \text{Eq. 8}$$

$$2.5\ HCO_3^- + 11.25\ H^+ + 10\ e^- \rightarrow 1.25\ CH_3COO^- + 5\ H_2O \qquad \text{Eq. 9}$$

$$(CH_3)_3N^+CH_2COO^- + H_2O + 0.5\ HCO_3^- \rightarrow 2.25\ CH_3COO^- + (CH_3)NH_3^+ + 0.75\ H^+ \qquad \text{Eq. 10}$$

$$(CH_3)_3N^+CH_2CH_2OH + H_2O \rightarrow (CH_3)_3N^+CH_2COO^- + 5\ H^+ + 4\ e^- \qquad \text{Eq. 11}$$

$$HCO_3^- + 4.5\ H^+ + 4\ e^- \rightarrow 0.5\ CH_3COO^- + 2\ H_2O \qquad \text{Eq. 12}$$

$$(CH_3)_3N^+CH_2CH_2OH + 1.5\ HCO_3^- \rightarrow CH_3NH_3^+ + 2.75\ CH_3COO^- + 1.25\ H^+ \qquad \text{Eq. 13}$$

Metabolism of methanol by DCMF

Methanol catabolism in DCMF is proposed to be carried out via a methanol methyltransferase system, which is similar to the three-component system described above for glycine betaine, comprising a methanol:corrinoid methyltransferase (MtaB, MT_1), methyl-tetrahydrofolate methyltransferase (MtaA, MT_2), and cognate corrinoid protein (MtaC). While such methanol methyltransferase systems are relatively well-described in methanogenic archaea (88–95), there are only a few reports from acetogenic bacteria, namely in *Moorella thermoacetica* (96), *S. ovata* (47) and *A. woodii* (97). The DCMF genome encodes a number of methanol-specific methyltransferases and associated corrinoid proteins (Table S5). Within the DCMF genome, the closest homolog to MtaB from *S. ovata* and *A. woodii* is a methanol:corrinoid methyltransferase (Ga0180325_112644). It resides in a cluster containing a methanol-specific MT_2 homolog (Ga0180325_112641) and a MtbC homolog (Ga0180325_112642 and Ga0180325_112645).

As in both *S. ovata* and *A. woodii*, the putative MT_2 gene in DCMF is a methyl-tetrahydrofolate methyltransferase, rather than the MtaA methanol methyltransferase found in methanogens (47,97). In *A. woodii*, the resulting methyl-tetrahydrofolate is then be transformed via the WLP (97) and DCMF is expected to follow a similar metabolic route, leading to the formation of acetate as the sole product.

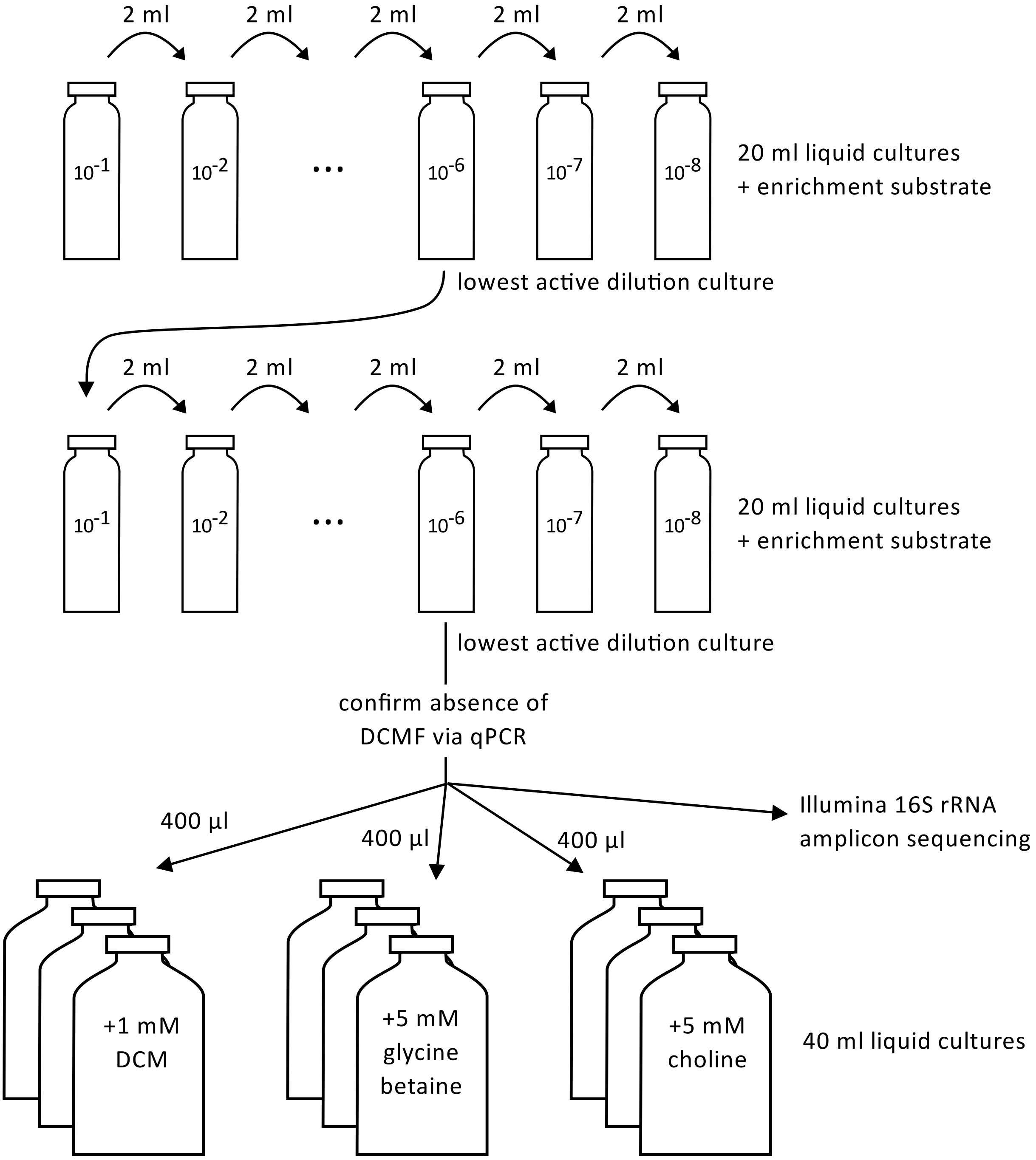

Supplementary Figure 1. Overview of the exclusion cultivation method used to generate DCMF-free cohabitant enrichment cultures.

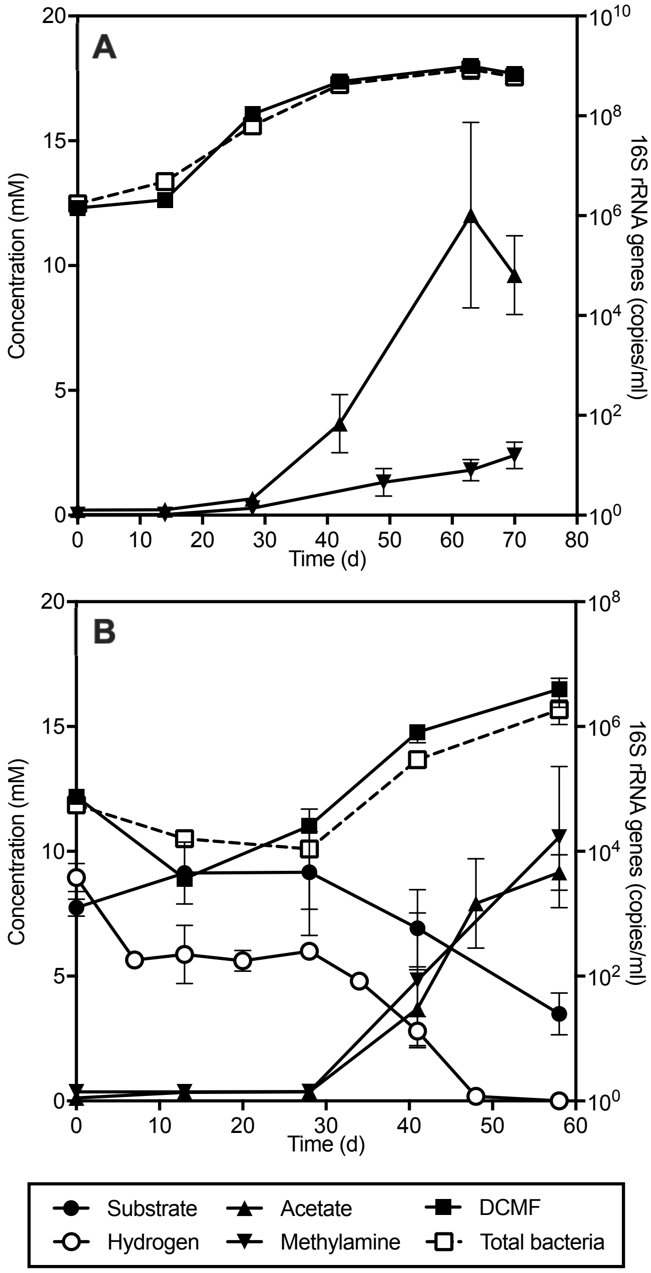

Supplementary Figure 2. Growth of DCMF with (A) dimethylglycine and (B) sarcosine (methylglycine) + H_2. Dimethylglycine could not be quantified. DCMF growth was concomitant with an increase in acetate and methylamine. Substrate and product concentrations are quantified on the left y-axis; DCMF and total bacterial 16S rRNA gene copies are quantified on the right y-axis. Error bars represent standard deviation, *n* = 3.

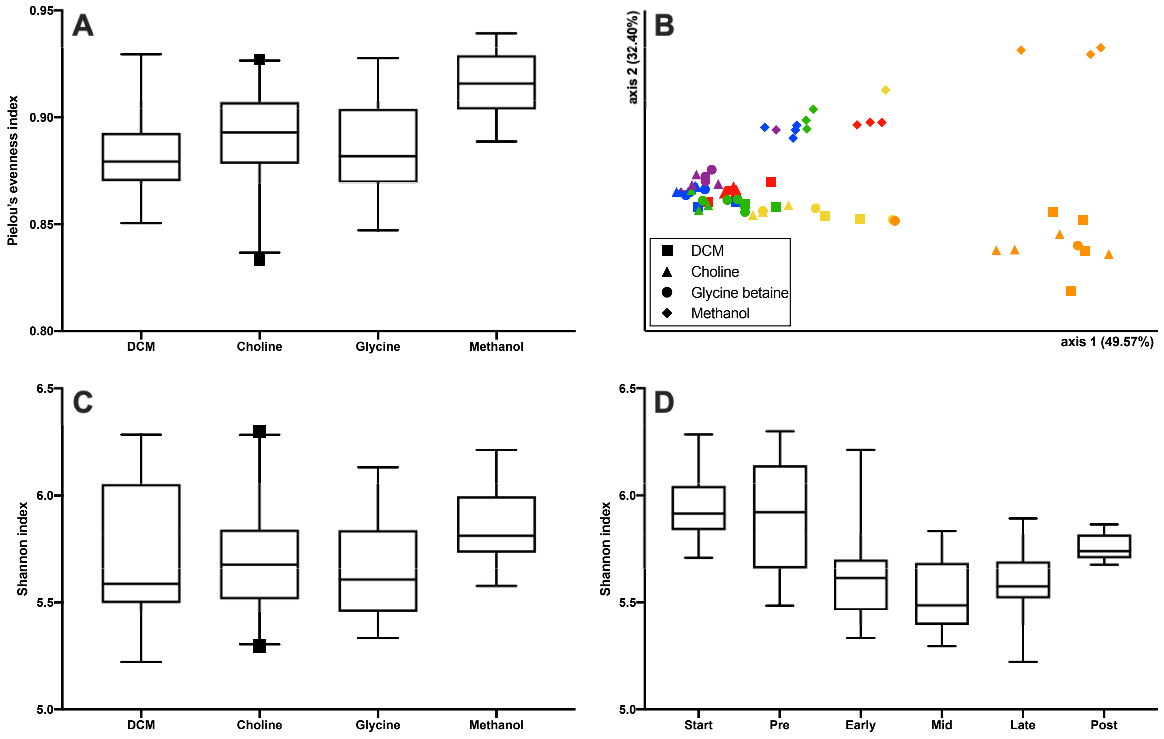

Supplementary Figure 3. Shifts in the culture DFE community are driven by the stage of substrate consumption more than difference in substrate. (A) Methanol-amended cultures had a significantly higher degree of evenness (adjusted p-value <0.01 in pairwise Kruskal-Wallis analysis of methanol compared to all other substrates) compared to cultures on the other three substrates, reflective of the lower relative abundance of DCMF in these cultures. (B) Principle components analysis plot of the weighted Unifrac distance matrix. Samples tended to group together based on substrate consumption proportion (colours) rather than differing substrate (shapes), although the methanol-amended community showed a higher degree of difference overall. Clusters emerged when samples were grouped by substrate consumption: inoculum/day 0 samples (red), pre (orange), early (yellow), mid (green), late (blue), or post (purple) (Table S3). There was no significant difference in the Shannon diversity index between samples grouped by substrate (C), but significant differences between samples when grouped by substrate consumption (D).

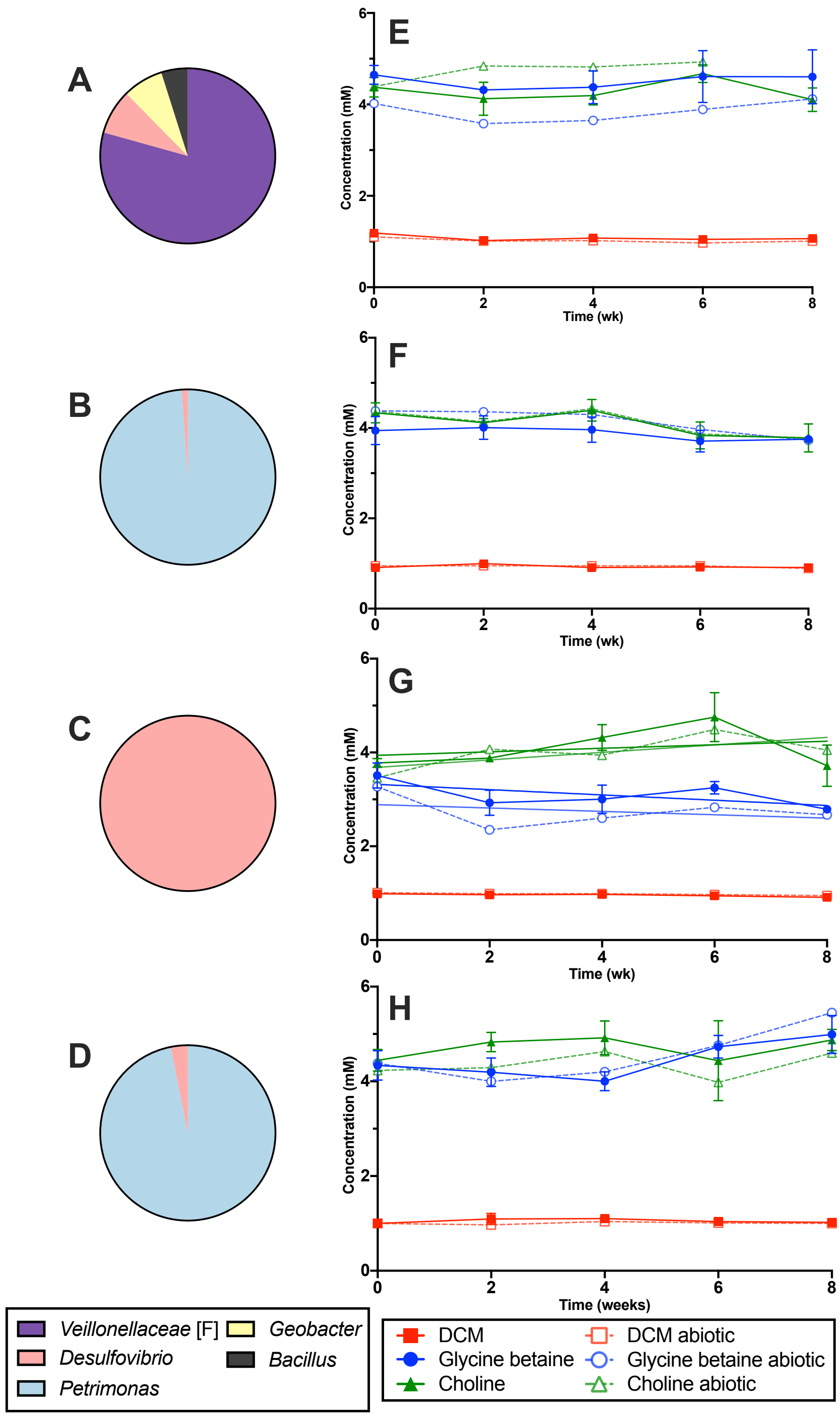

Supplementary Figure 4. DCMF-free DFE community enrichments amended with DCM, glycine betaine, or choline. Community profiling of the lowest active dilution culture in the second round of dilution-to-extinction DFE cultures amended with casamino acids (A), glucose (B), peptone (C), or yeast extract (D). These DCMF-free enrichments were then transferred back into medium containing the typical DCMF substrates (DCM, glycine betaine, choline) (cultures A-D represent the inocula for cultures depicted in E-H, respectively). Error bars represent standard deviation; active cultures $n = 3$, abiotic $n = 1$.

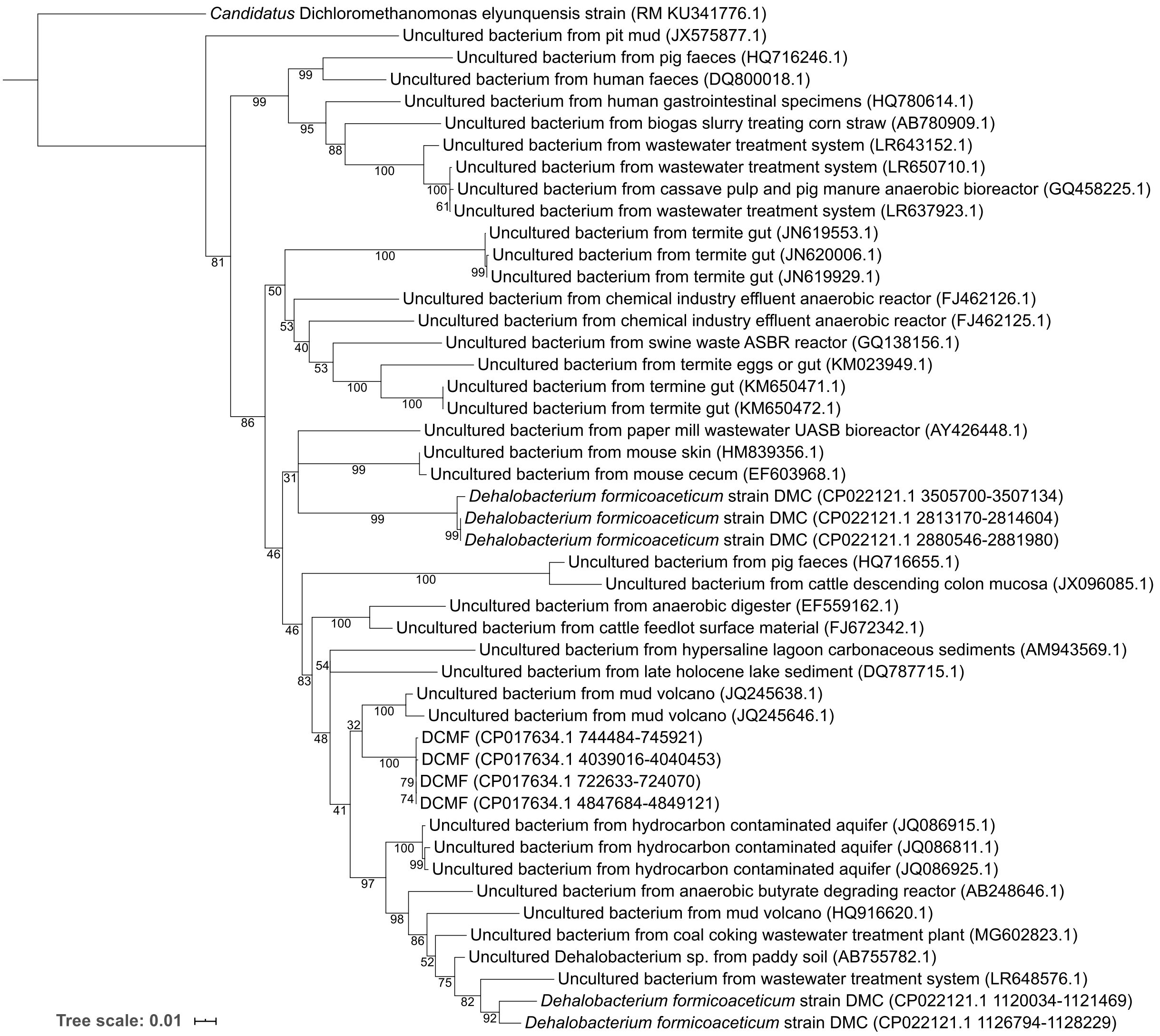

Supplementary Figure 5. Phylogenetic tree of the DCMF 16S rRNA genes and closely related sequences. The DCMF 16S rRNA gene consensus sequence was searched against the NCBI nr database using BLAST. All sequences with ≥90% nucleotide identity were manually trimmed and aligned with MUSCLE. A phylogenetic tree was inferred by IQ-TREE v1.6.1 (98) using ModelFinder (99) with ultrafast bootstrapping (1000). The tree was rooted with the ‘*Ca.* Dichloromethanomonas elyunquensis’ strain RM 16S rRNA gene sequence.

Supplementary Table 1. Analytical methods used to quantify bicarbonate, hydrogen, methanol, methylated/quaternary amines, organohalides, volatile fatty acids via gas chromatography.

See Supplementary Tables Excel document.

Supplementary Table 2. PCR and qPCR primer pairs and amplification conditions used in this study.

**Target**	**Primer Pair (sequence 5' to 3')**	**Reaction mixture**	**Protocol**	**Reference**
Bacterial 16S rRNA gene (PCR for Illumina amplicon sequencing)	515 (TCGTCGGCAGCGTCAGATGTGTATAAGAGAC-AGGTGYCAGCMGCCGCGGTAA)<br>806 (GTCTCGTGGGCTCGGAGATGTGTATAAGAGA-CAGGGACTACNVGGGTWTCTAAT)	2 µl template DNA, 10 µl EconoTaq PLUS Green 2X master mix (Lucigen), 100 nM of forward and reverse primers (0.4 µl each of 10 µM stocks; IDT), 17.2 µl molecular grade water (Sigma).	1) 94˚C, 3:00; 2) 94˚C, 0:45; 3) 50˚C, 1:00; 4) 72˚C, 1:30; 5) go to step 2, 34 times; 6) 72˚C, 10:00	(100)
Bacterial 16S rRNA gene (qPCR)	Eub1084 (GTGSTGCAYGGYTGTCGTCA)<br>Eub1194 (ACGTCRTCCMCACCTTCCTC)	2 µl template DNA, 5 µl 2X SsoFast™ EvaGreen® Supermix (Bio-Rad), 100 nM of forward and reverse primers (0.1 µl each of 10 µM stocks; IDT), 0.1 µl bovine serum albumin (10 mg ml^{-1}), 2.7 µl molecular grade water (Sigma).	1) 98˚C, 3:00; 2) 95˚C, 0:20; 3) 62˚C; 0:50 4) acquisition of SYBR channel; 5) go to Step 2, 39 times	(26)
DCMF 16S rRNA gene (qPCR)	Dcm775 (AAGGCGACTTTCTGGACTGA)<br>Dcm930 (GCGGGGTACTTATTGCGTTA)		1) 98˚C, 3:00; 2) 94˚C, 1:00; 3) 60˚C; 0:45 4) acquisition of SYBR channel; 5) go to Step 2, 39 times	This study

Supplementary Table 3. Classification of Illumina 16S rRNA gene amplicon sequencing samples in relation to the amount of substrate consumed.

Classification	Substrate	Time (d)	Replicate
Start	DCM	0	Inoculum
		0	A, B
	Glycine betaine	0	A, B, C
	Choline	0	A, B, C
	Methanol	0	A, B, C
Pre	DCM	14, 21	A, B
	Glycine betaine	7	A, B
	Choline	7	A, B, C
		11	A
	Methanol	14	A, C
		19	A
Early	DCM	25	A, B
	Glycine betaine	7	C
		11	A, B
	Choline	11	B, C
		15	A
	Methanol	23	A
Mid	DCM	29	A, B
	Glycine betaine	11	C
		15	A, B
	Choline	15	B, C
		21	A
	Methanol	14	B
		23	C
		26	A
Late	DCM	35	A, B
	Glycine betaine	21	A, B, C
	Choline	21	B, C
		25	A, B
		28	A
	Methanol	19	B
		26	C
		30	A, C
Post	Glycine betaine	28	A, B, C
	Choline	25	C
		28	B, C
	Methanol	26	B

Supplementary Table 4. ^{13}C mass balances for experiments with ^{13}C-labelled DCM and bicarbonate. All values are in µM.

**Labelled compound**	**Input**	**Output**							
	**[^{13}C]**	**[1-^{13}C]acetate**	**[2-^{13}C]acetate**	**[1,2-^{13}C]acetate [a]**	**$H^{13}CO_3^-{}_{(aq)}$**	**$^{13}CO_{2\,(g)}$ [b]**	**^{13}C acetate equivalents in biomass[c]**	**Total output[d]**	**Recovery[c]**
**[^{13}C]DCM**	2 700 ± 328	0	266 ± 92.8	202 ± 99.9	815 ± 120	982 ± 144	49.7 ± 6.0	2 320 ± 432	128% ± 8.2%
**$H^{13}CO_3^-$**	7 170 ± 441	454 ± 51.0	24 ± 14.8	97 ± 13.4	2 278 ± 170	2 744 ± 204	710 ± 9.74	6 400 ± 370	84.5% ± 6.98%

[a]Values are for the amount of labelled carbon in [1,2-13C]acetate, i.e. twice the measured concentration of [1,2-13C]acetate, as it contains two labelled carbons.

[b]Calculated based on the Henry's Law dimensionless volatility constant (H_{cc} = 1.20 at 25˚C).

[c]Acetate equivalents in biomass were calculated as detailed in the Methods section.

[d]Totals and recovery percentages were calculated for each replicate (n = 3) separately and then averaged, hence the values do not add up directly when calculated from this table.

Supplementary Table 5. Genes involved in the metabolic model presented in Figure 6.

See Supplementary Tables Excel document.

Supplementary Information References

62. Duhamel M, Edwards EA. Microbial composition of chlorinated ethene-degrading cultures dominated by *Dehalococcoides*. FEMS Microbiol Ecol. 2006 Dec;58(3):538–49.

63. Kleinsteuber S, Schleinitz KM, Breitfeld J, Harms H, Richnow HH, Vogt C. Molecular characterization of bacterial communities mineralizing benzene under sulfate-reducing conditions. FEMS Microbiol Ecol. 2008;66(1):143–57.

64. Strąpoć D, Mastalerz M, Dawson K, Macalady J, Callaghan A V., Wawrik B, et al. Biogeochemistry of microbial coal-bed methane. Annu Rev Earth Planet Sci. 2011;39(1):617–56.

65. Taubert M, Vogt C, Wubet T, Kleinsteuber S, Tarkka MT, Harms H, et al. Protein-SIP enables time-resolved analysis of the carbon flux in a sulfate-reducing, benzene-degrading microbial consortium. ISME J. 2012;6(12):2291–301.

66. Dong X, Greening C, Brüls T, Conrad R, Guo K, Blaskowski S, et al. Fermentative Spirochaetes mediate necromass recycling in anoxic hydrocarbon-contaminated habitats. ISME J. 2018;12(8):2039–50.

67. Kleinsteuber S, Schleinitz KM, Vogt C. Key players and team play: Anaerobic microbial communities in hydrocarbon-contaminated aquifers. Appl Microbiol Biotechnol. 2012;94(4):851–73.

68. Jumas-Bilak E, Carlier JP, Jean-Pierre H, Citron D, Bernard K, Damay A, et al. *Jonquetella anthropi* gen. nov., sp. nov., the first member of the candidate phylum "*Synergistetes*" isolated from man. Int J Syst Evol Microbiol. 2007;57(12):2743–8.

69. Pitluck S, Yasawong M, Held B, Lapidus A, Nolan M, Copeland A, et al. Non-contiguous finished genome sequence of *Aminomonas paucivorans* type strain (GLU-3 T). Stand Genomic Sci. 2010;3(3):285–93.

70. Vartoukian SR, Palmer RM, Wade WG. The division "*Synergistes*." Anaerobe. 2007;13(3–4):99–106.

71. Einsiedl F, Pilloni G, Ruth-Anneser B, Lueders T, Griebler C. Spatial distributions of sulphur species and sulphate-reducing bacteria provide insights into sulphur redox cycling and biodegradation hot-spots in a hydrocarbon-contaminated aquifer. Geochim Cosmochim Acta. 2015;156:207–21.

72. Tan B, Jane Fowler S, Laban NA, Dong X, Sensen CW, Foght J, et al. Comparative analysis of metagenomes from three methanogenic hydrocarbon-degrading enrichment cultures with 41 environmental samples. ISME J. 2015;9(9):2028–45.

73. Marchandin H, Jumas-Bilak E. The Family Veillonellaceae. In: Rosenberg E, Delong EF, Thompson F, editors. The Prokaryotes: Firmicutes and Tenericutes. Berlin: Springer-Verlag; 2014.

74. Grabowski A, Tindall BJ, Bardin V, Blanchet D, Jeanthon C. *Petrimonas sulfuriphila* gen. nov., sp. nov., a mesophilic fermentative bacterium isolated from a biodegraded oil reservoir. Int J Syst Evol Microbiol. 2005;55(3):1113–21.

75. Hahnke S, Langer T, Koeck DE, Klocke M. Description of *Proteiniphilum saccharofermentans* sp. nov., *Petrimonas mucosa* sp. nov. and *Fermentimonas caenicola* gen. nov., sp. nov., isolated from mesophilic laboratory-scale biogas reactors, and emended description of the genus *Proteiniphilum*. Int J Syst Evol Microbiol. 2016;66(3):1466–75.

76. Sun L, Toyonaga M, Ohashi A, Tourlousse DM, Matsuura N, Meng XY, et al. *Lentimicrobium saccharophilum* gen. nov., sp. nov., a strictly anaerobic bacterium representing a new family in the phylum *Bacteroidetes*, and proposal of *Lentimicrobiaceae* fam. nov. Int J Syst Evol Microbiol. 2016;66(7):2635–42.

77. Löffler FE, Sanford R a, Ritalahti KM. Enrichment, cultivation, and detection of reductively dechlorinating bacteria. Methods Enzymol. 2005 Jan;397(1996):77–111.

78. Müller S, Vogt C, Laube M, Harms H, Kleinsteuber S. Community dynamics within a bacterial consortium during growth on toluene under sulfate-reducing conditions. FEMS Microbiol Ecol. 2009;70(3):586–96.

79. Welsh DT. Ecological significance of compatible solute accumulation by micro-organisms: from single cells to global climate. FEMS Microbiol Rev. 2000;24(3):263–90.

80. Craciun S, Balskus EP. Microbial conversion of choline to trimethylamine requires a glycyl radical enzyme. Proc Natl Acad Sci. 2012;109(52):21307–12.

81. Watkins AJ, Roussel EG, Webster G, Parkes RJ, Sass H. Choline and N,N-dimethylethanolamine as direct substrates for methanogens. Appl Environ Microbiol. 2012;78(23):8298–303.

82. Müller E, Fahlbusch K, Walther R, Gottschalk G. Formation of N,N-dimethylglycine, acetic

acid, and butyric acid from betaine by *Eubacterium limosum*. Appl Environ Microbiol. 1981;42(3):439–45.

83. Eichler B, Schink B. Oxidation of primary aliphatic alcohols by *Acetobacterium carbinolicum* sp. nov., a homoacetogenic anaerobe. Arch Microbiol. 1984;140(2–3):147–52.

84. Tanaka K, Pfennig N. Fermentation of 2-methoxyethanol by *Acetobacterium malicum* sp. nov. and *Pelobacter venetianus*. Arch Microbiol. 1988;149(3):181–7.

85. Kotsyurbenko OR, Simankova M V., Nozhevnikova AN, Zhilina TN, Bolotina NP, Lysenko AM, et al. New species of psychrophilic acetogens: *Acetobacterium bakii* sp. nov., *A. paludosum* sp. nov., *A. fimetarium* sp. nov. Arch Microbiol. 1995;163(1):29–34.

86. Ticak T, Kountz DJ, Girosky KE, Krzycki JA, Ferguson DJ. A nonpyrrolysine member of the widely distributed trimethylamine methyltransferase family is a glycine betaine methyltransferase. Proc Natl Acad Sci U S A. 2014;111(43):E4668–76.

87. Lechtenfeld M, Heine J, Sameith J, Kremp F, Müller V. Glycine betaine metabolism in the acetogenic bacterium *Acetobacterium woodii*. Environ Microbiol. 2018 Dec 5;20(12):4512–25.

88. van der Meijden P, Jansen LPJM, Drift C, Vogels GD. Involvement of corrinoids in the methylation of coenzyme M (2-mercaptoethanesulfonic acid) by methanol and enzymes from *Methanosarcina barkeri*. FEMS Microbiol Lett. 1983;19(2–3):247–51.

89. van der Meijden P, Heythuysen HJ, Pouwels A, Houwen F, van der Drift C, Vogels GD. Methyltransferases involved in methanol conversion by *Methanosarcina barkeri*. Arch Microbiol. 1983;134(3):238–42.

90. van der Meijden P, Te Brömmelstroet BW, Poirot CM, van der Drift C, Vogels GD. Purification and properties of methanol:5-hydroxybenzimidazolylcobamide methyltransferase from *Methanosarcina barkeri*. J Bacteriol. 1984;160(2):629–35.

91. Burke SA, Krzycki JA. Involvement of the "A" isozyme of methyltransferase II and the 29-kilodalton corrinoid protein in methanogenesis from monomethylamine. J Bacteriol. 1995;177(15):4410–6.

92. Burke SA, Krzycki JA. Reconstitution of monomethylamine:coenzyme M methyl transfer with a corrinoid protein and two methyltransferases purified from *Methanosarcina*

barkeri. J Biol Chem. 1997;275(37):29053–60.

93. Sauer K, Thauer RK. Methanol:coenzyme M methyltransferase from *Methanosarcina barkeri*: Zinc dependence and thermodynamics of the methanol:cob(I)alamin methyltransferase reaction. Eur J Biochem. 1997;249:280–5.

94. Sauer K, Harms U, Thauer RK. Methanol:coenzyme M methyltransferase from *Methanosarcina barkeri*: purification, properties and encoding genes of the corrinoid protein MT1. Eur J Biochem. 1997;243(3):670–7.

95. Hagemeier CH, Krüer M, Thauer RK, Warkentin E, Ermler U. Insight into the mechanism of biological methanol activation based on the crystal structure of the methanol-cobalamin methyltransferase complex. Proc Natl Acad Sci U S A. 2006;103(50):18917–22.

96. Das A, Fu Z-Q, Tempel W, Liu Z-J, Chang J, Chen L, et al. Characterization of a corrinoid protein involved in the C1 metabolism of strict anaerobic bacterium *Moorella thermoacetica*. Proteins Struct Funct Bioinforma. 2007 Jan 8;67(1):167–76.

97. Kremp F, Poehlein A, Daniel R, Müller V. Methanol metabolism in the acetogenic bacterium *Acetobacterium woodii*. Environ Microbiol. 2018;20(12):4369–84.

98. Nguyen LT, Schmidt HA, Von Haeseler A, Minh BQ. IQ-TREE: A fast and effective stochastic algorithm for estimating maximum-likelihood phylogenies. Mol Biol Evol. 2015;32(1):268–74.

99. Kalyaanamoorthy S, Minh BQ, Wong TKF, von Haeseler A, Jermiin LS. ModelFinder: fast model selection for accurate phylogenetic estimates. Nat Methods. 2017;14(6):587–9.

100. Caporaso JG, Lauber CL, Walters WA, Berg-Lyons D, Lozupone CA, Turnbaugh PJ, et al. Global patterns of 16S rRNA diversity at a depth of millions of sequences per sample. PNAS. 2011;108(suppl. 1):4516–22.
